# 1-(Morpholin-4-yl)-4-(2-nitro­phen­yl)spiro­[azetidine-3,9′-xanthen]-2-one

**DOI:** 10.1107/S1600536814013464

**Published:** 2014-06-14

**Authors:** Zeliha Atioğlu, Mehmet Akkurt, Aliasghar Jarrahpour, Roghayeh Heiran, Namık Özdemir

**Affiliations:** a Ilke Education and Health Foundation, Cappadocia Vocational College, The Medical Imaging Techniques Program, 50420 Mustafapaşa, Ürgüp, Nevşehir, Turkey; b Department of Physics, Faculty of Sciences, Erciyes University, 38039 Kayseri, Turkey; c Department of Chemistry, College of Sciences, Shiraz University, 71454 Shiraz, Iran; d Department of Physics, Faculty of Arts and Sciences, Ondokuz Mayıs University, 55139 Samsun, Turkey

## Abstract

In the title compound, C_22_H_21_N_3_O_5_, the β-lactam (azetidin-2-one) ring is nearly planar [maximum deviation = 0.010 (1) Å] and makes dihedral angles of 69.22 (5), 55.32 (5) and 89.42 (4)° with the least-squares planes formed by the four C atoms of the morpholine ring, which adopts a chair conformation, the benzene ring and the xanthene ring system, respectively. In the crystal, C—H⋯O hydrogen-bond contacts connect neighbouring mol­ecules into infinite zigzag chains running parallel to the *b* axis.

## Related literature   

For general background to β-lactams, see: Arya *et al.* (2014 ▶); Ebrahimi & Jarrahpour (2014 ▶); Singh & Sudheesh (2014 ▶); Zeng *et al.* (2014 ▶); Zarei *et al.* (2013 ▶); Jarrahpour & Ebrahimi (2010 ▶); Mehta *et al.* (2010 ▶); Singh *et al.* (2011 ▶). For geometric analysis, see: Cremer & Pople (1975 ▶); Nardelli (1995 ▶). For similar structures, see: Akkurt *et al.* (2008*a* ▶,*b* ▶); Yalçın *et al.* (2009 ▶); Çelik *et al.* (2009*a* ▶,*b* ▶, 2014 ▶). 
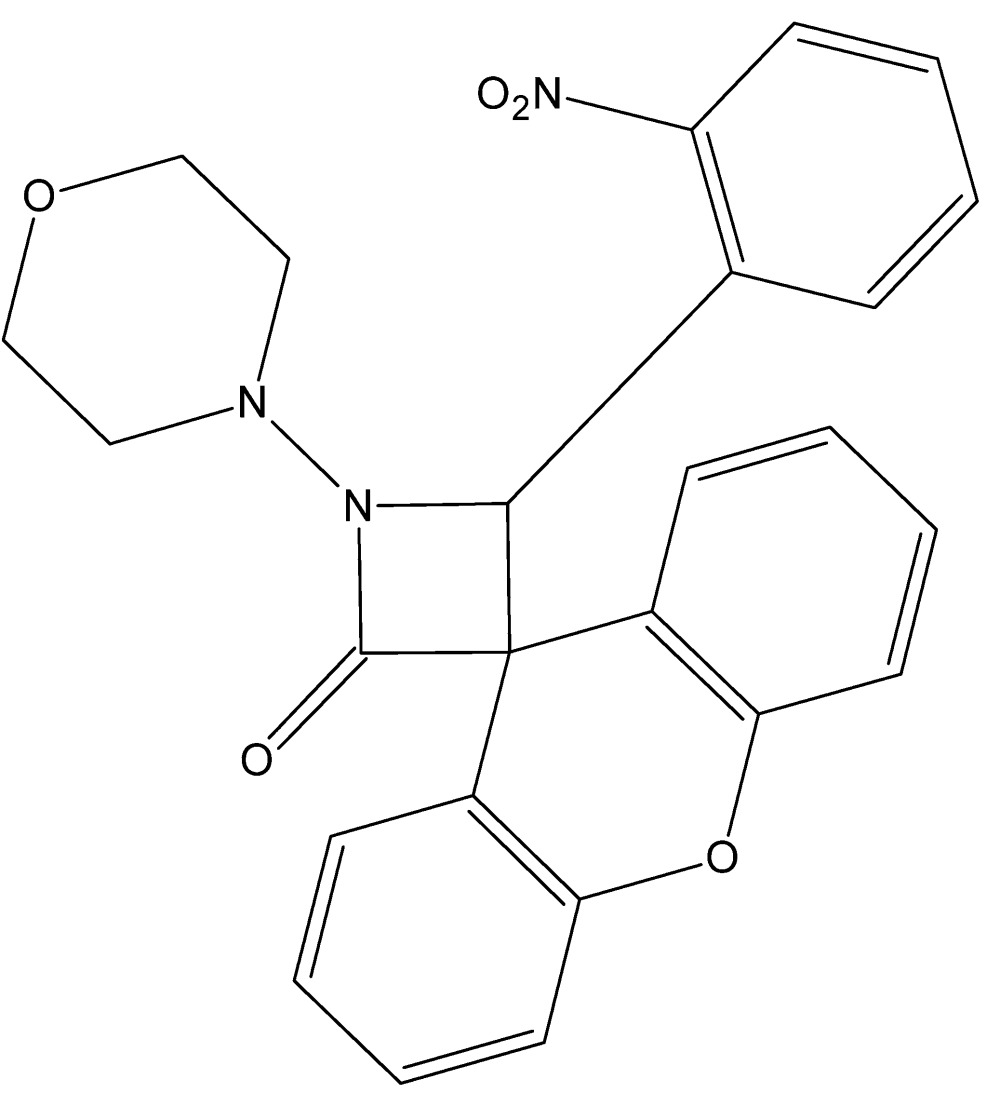


## Experimental   

### 

#### Crystal data  

C_25_H_21_N_3_O_5_
*M* *_r_* = 443.45
Monoclinic, 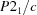

*a* = 9.4272 (5) Å
*b* = 18.8525 (8) Å
*c* = 12.4345 (6) Å
β = 95.443 (4)°
*V* = 2199.97 (18) Å^3^
*Z* = 4
Mo *K*α radiation
μ = 0.10 mm^−1^
*T* = 296 K
0.50 × 0.44 × 0.40 mm

#### Data collection  

Stoe IPDS 2 diffractometer
Absorption correction: integration (*X-RED32*; Stoe & Cie, 2002 ▶) *T*_min_ = 0.956, *T*_max_ = 0.974
13801 measured reflections
5223 independent reflections
3421 reflections with *I* > 2σ(*I*)
*R*_int_ = 0.195

#### Refinement  

*R*[*F* ^2^ > 2σ(*F* ^2^)] = 0.045
*wR*(*F* ^2^) = 0.098
*S* = 1.00
5223 reflections
299 parameters
H-atom parameters constrained
Δρ_max_ = 0.15 e Å^−3^
Δρ_min_ = −0.11 e Å^−3^

### 

Data collection: *X-AREA* (Stoe & Cie, 2002 ▶); cell refinement: *X-AREA*; data reduction: *X-RED32* (Stoe & Cie, 2002 ▶); program(s) used to solve structure: *SHELXS2013* (Sheldrick, 2008 ▶); program(s) used to refine structure: *SHELXL2013* (Sheldrick, 2008 ▶); molecular graphics: *ORTEP-3 for Windows* (Farrugia, 2012 ▶); software used to prepare material for publication: *WinGX* (Farrugia, 2012 ▶).

## Supplementary Material

Crystal structure: contains datablock(s) global, I. DOI: 10.1107/S1600536814013464/sj5411sup1.cif

Structure factors: contains datablock(s) I. DOI: 10.1107/S1600536814013464/sj5411Isup2.hkl

Supporting information file. DOI: 10.1107/S1600536814013464/sj5411Isup3.cml

CCDC reference: 1007508

Additional supporting information:  crystallographic information; 3D view; checkCIF report

## Figures and Tables

**Table 1:** Hydrogen-bond geometry (Å, °)

*D*—H⋯*A*	*D*—H	H⋯*A*	*D*⋯*A*	*D*—H⋯*A*
C3—H3⋯O3^i^	0.98	2.55	3.5310 (16)	174
C6—H6⋯O1^ii^	0.93	2.56	3.3828 (17)	148
C11—H11⋯O2^iii^	0.93	2.50	3.389 (2)	159

Symmetry codes: (i) 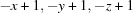; (ii) 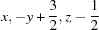; (iii) 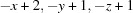.

**Fig. 1.**
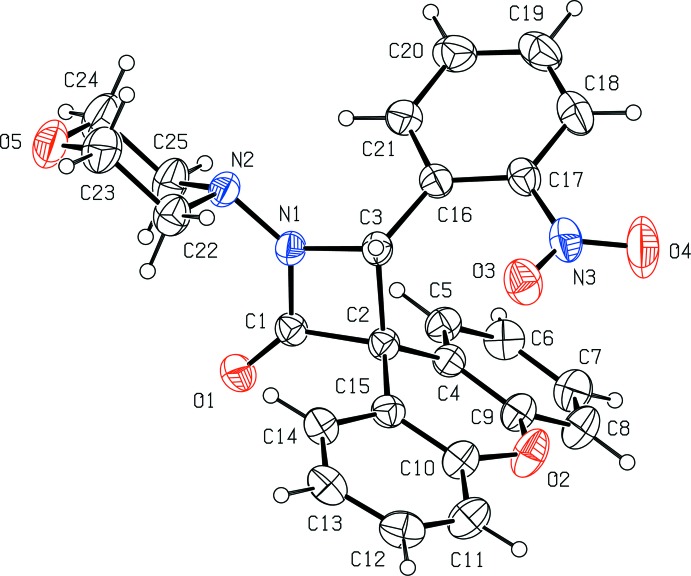
The molecular structure of (I) with the atom numbering scheme. Displacement ellipsoids for non-H atoms are drawn at the 30% probability level.

**Fig. 2.**
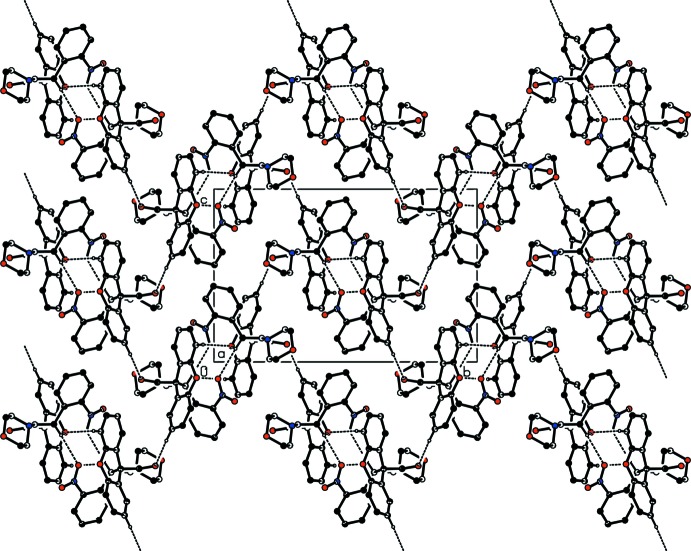
Hydrogen bonding and molecular packing of (I) viewed along the a axis. Only H atoms involved in H bonding are shown.

**Fig. 3.**
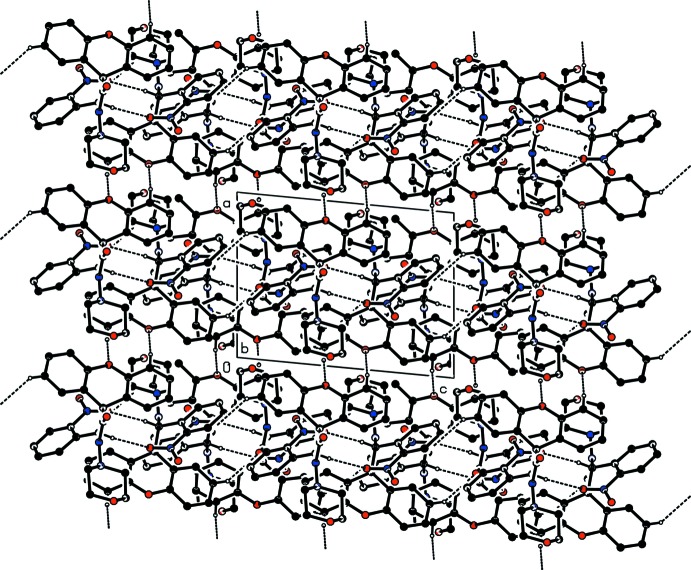
Hydrogen bonding and molecular packing of (I) viewed along the b axis. Only H atoms involved in H bonding are shown.

**Fig. 4.**
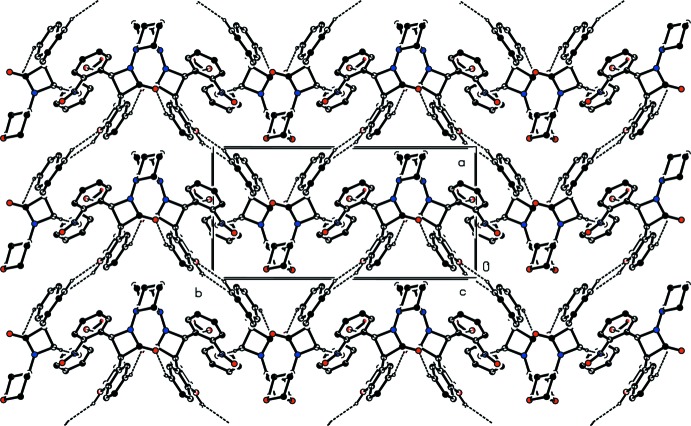
Hydrogen bonding and molecular packing of (I) viewed along the c axis. Only H atoms involved in H bonding are shown.

## supplementary crystallographic information

### S1. Comment

2-Azetidinones, commonly known as β-lactams, constitute a most important class of antibiotics in both human and veterinary medicine (Arya *et al.*, 2014; Singh & Sudheesh, 2014; Zeng *et al.*, 2014; Zarei *et al.*, 2013). In addition to their well recognized antibiotic activity, β-lactams exhibit various other biological activities such as thrombin, human, HIV-1 protease, human leukocyte elastase, cholesterol absorption inhibition and antifungal, anticancer, antidiabetic and potential antimalarial properties (Mehta *et al.*, 2010; Singh *et al.*, 2011; Ebrahimi & Jarrahpour, 2014). The synthesis and chemistry of spiro-fused 2-azetidinones has grown steadily over the years and many newly synthesized spiro-fused 2-azetidinones have been reported in the literature (Jarrahpour & Ebrahimi, 2010; Singh *et al.*, 2011).

The β-lactam (azetidin-2-one) ring of the title compound (I, Fig. 1) is nearly planar, with a maximum deviation of -0.010 (1) Å for C1 from the mean plane. Atom O1 lies almost in the β-lactam plane, with a deviation of 0.069 (1) Å. The β-lactam ring makes a dihedral angle of 55.32 (5)° with the benzene ring C16—C21.

The xanthene ring system is V-shaped, with a dihedral angle between the (C4–C9) and (C10–C15) benzene rings of 19.07 (7)°. Its central ring, C2/C4/C9/O2/C10/C15, is not planar, with puckering parameters: Q_T_ = 0.2438 (13) Å, θ = 98.1 (3)° and φ = 2.0 (3)° (Cremer & Pople, 1975).

The mean plane of the xanthene ring system forms dihedral angles of 89.42 (4), 43.44 (3) and 22.80 (5)° (Nardelli, 1995), with the β-lactam ring, the benzene ring (C16–C21) and the least-squares plane formed by the four C atoms of the morpholine ring (N2/O5/C22–C25), respectively.

The bond lengths and angles in (I) are comparable with those observed in similar compounds that we have reported previously (Akkurt *et al.*, 2008*a*,*b*; Çelik *et al.*, 2009*a*,*b*; Çelik *et al.*, 2014; Yalçın *et al.*, 2009).

In the crystal structure, molecules are linked by C—H···O hydrogen contacts (Table 1) into infinite zigzag chains running parallel to the *b* axis. Figs. 2, 3 and 4 show the projections along the *a, b and c* axes of the crystal packing of (I), respectively.

### S2. Experimental

A mixture of *N*-(2-nitrobenzylidene)morpholin-4-amine (0.24 g, 1.00 mmol), 9*H*-xanthen-9-carboxylic acid (0.34 g, 1.50 mmol), tosyl chloride (0.28 g, 1.50 mmol) and triethylamine (0.25 g, 2.50 mmol) was stirred in dry CH_2_Cl_2_ at room temperature. After 24 h, the mixture was washed with HCl 1 M (20 ml), saturated NaHCO_3_ (20 ml), brine (20 ml), dried over Na_2_SO_4_ and the solvent was evaporated to give the crude product which was purified by column chromatography (eluent 2:1 n-hexane/EtOAc) as light yellow crystalline solid (yield 41%). mp: 471- 473 K. IR (KBr, cm^-1^): 1759 (CO, β-lactam), 1346, 1523 (NO2). ^1^H-NMR (CDCl3) δ (p.p.m.): 3.52–3.76 (CH_2_ morpholine ring, m, 8H), 5.38 (H-3, s, 1H), 6.62–8.10 (ArH, m, 12H). ^13^C-NMR (CDCl_3_) δ (p.p.m.): 53.8 (CH_2_—N), 61.4 (C-3), 66.8 (CH_2_—O), 73.9 (C-4), 114.9, 116.8, 116.9, 120.5, 122.2, 123.9, 124.8, 125.1, 127.8, 128.9, 129.3, 129.5, 131.1, 133.1, 147.5, 152.1, 152.3 (aromatic carbons), 169.7 (CO, β-lactam). Anal. calcd for C_25_H_21_N_3_O_5_: C 67.71, H 4.77, N 9.48%. Found: C 67.80, H 4.66, N 9.45%.

### S3. Refinement

All H atoms were positioned geometrically and were refined using a riding model, with C—H = 0.93 (aromatic), 0.97 Å (methylene) 0.98 Å(methine), respectively, and *U*_iso_(H) = 1.2 *U*_eq_(C). Reflections (1 4 1), (0 3 2), (-1 2 2), (-2 0 2), (1 3 0) and (1 5 0) were omitted due to the large disagreement between F_obs_ and F_calc_. Owing to the poor quality of the crystal, the data obtained were rather poor and the value of *R*_int_ remained high (0.195).

### Crystal data

**Table:**

C_25_H_21_N_3_O_5_	*F*(000) = 928
*M**_r_* = 443.45	*D*_x_ = 1.339 Mg m^−^^3^
Monoclinic, *P*2_1_/*c*	Mo *K*α radiation, λ = 0.71073 Å
Hall symbol: -P 2ybc	Cell parameters from 14473 reflections
*a* = 9.4272 (5) Å	θ = 1.6–28.4°
*b* = 18.8525 (8) Å	µ = 0.10 mm^−^^1^
*c* = 12.4345 (6) Å	*T* = 296 K
β = 95.443 (4)°	Block, light yellow
*V* = 2199.97 (18) Å^3^	0.50 × 0.44 × 0.40 mm
*Z* = 4	

### Data collection

**Table:**

Stoe IPDS 2 diffractometer	5223 independent reflections
Radiation source: sealed X-ray tube, 12 x 0.4 mm long-fine focus	3421 reflections with *I* > 2σ(*I*)
Plane graphite monochromator	*R*_int_ = 0.195
Detector resolution: 6.67 pixels mm^-1^	θ_max_ = 27.9°, θ_min_ = 2.0°
ω scans	*h* = −12→8
Absorption correction: integration (*X-RED32*; Stoe & Cie, 2002)	*k* = −24→24
*T*_min_ = 0.956, *T*_max_ = 0.974	*l* = −16→16
13801 measured reflections	

### Refinement

**Table:**

Refinement on *F*^2^	0 restraints
Least-squares matrix: full	Hydrogen site location: inferred from neighbouring sites
*R*[*F*^2^ > 2σ(*F*^2^)] = 0.045	H-atom parameters constrained
*wR*(*F*^2^) = 0.098	*w* = 1/[σ^2^(*F*_o_^2^) + (0.0511*P*)^2^] where *P* = (*F*_o_^2^ + 2*F*_c_^2^)/3
*S* = 1.00	(Δ/σ)_max_ < 0.001
5223 reflections	Δρ_max_ = 0.15 e Å^−^^3^
299 parameters	Δρ_min_ = −0.11 e Å^−^^3^

### Special details

**Table:**

Geometry. Bond distances, angles *etc*. have been calculated using the rounded fractional coordinates. All su's are estimated from the variances of the (full) variance-covariance matrix. The cell e.s.d.'s are taken into account in the estimation of distances, angles and torsion angles
Refinement. Refinement on *F*^2^ for ALL reflections except those flagged by the user for potential systematic errors. Weighted *R*-factors *wR* and all goodnesses of fit *S* are based on *F*^2^, conventional *R*-factors *R* are based on *F*, with *F* set to zero for negative *F*^2^. The observed criterion of *F*^2^ > σ(*F*^2^) is used only for calculating -*R*-factor-obs *etc*. and is not relevant to the choice of reflections for refinement. *R*-factors based on *F*^2^ are statistically about twice as large as those based on *F*, and *R*-factors based on ALL data will be even larger.

### Fractional atomic coordinates and isotropic or equivalent isotropic displacement parameters (Å^2^)

**Table:**

	*x*	*y*	*z*	*U*_iso_*/*U*_eq_	
O1	0.56523 (11)	0.77674 (4)	0.39775 (8)	0.0607 (3)	
O2	0.88039 (12)	0.56659 (6)	0.40826 (8)	0.0725 (4)	
O3	0.59829 (13)	0.48451 (5)	0.40169 (9)	0.0738 (4)	
O4	0.6611 (2)	0.42173 (8)	0.27221 (13)	0.1293 (7)	
O5	0.05898 (13)	0.80037 (7)	0.43054 (10)	0.0862 (5)	
N1	0.40654 (11)	0.68165 (5)	0.36241 (8)	0.0475 (3)	
N2	0.26494 (11)	0.70325 (5)	0.37024 (8)	0.0504 (3)	
N3	0.58819 (15)	0.46781 (6)	0.30725 (11)	0.0673 (5)	
C1	0.53326 (14)	0.71537 (6)	0.38183 (9)	0.0459 (4)	
C2	0.61723 (13)	0.64499 (6)	0.38092 (9)	0.0439 (4)	
C3	0.46359 (13)	0.60880 (6)	0.36101 (9)	0.0444 (4)	
C4	0.71522 (13)	0.63736 (6)	0.29281 (9)	0.0450 (4)	
C5	0.68634 (14)	0.66829 (7)	0.19146 (10)	0.0527 (4)	
C6	0.77624 (16)	0.65986 (8)	0.11144 (11)	0.0610 (5)	
C7	0.89724 (16)	0.61957 (8)	0.13129 (12)	0.0648 (5)	
C8	0.92974 (16)	0.58875 (8)	0.23033 (12)	0.0665 (5)	
C9	0.83854 (14)	0.59821 (7)	0.31040 (10)	0.0536 (4)	
C10	0.81733 (15)	0.58925 (7)	0.49807 (10)	0.0554 (4)	
C11	0.88545 (17)	0.56948 (9)	0.59700 (12)	0.0697 (6)	
C12	0.83068 (17)	0.59109 (8)	0.68992 (12)	0.0668 (5)	
C13	0.71169 (18)	0.63341 (7)	0.68493 (11)	0.0631 (5)	
C14	0.64380 (16)	0.65115 (7)	0.58589 (10)	0.0555 (4)	
C15	0.69405 (14)	0.62872 (6)	0.48998 (10)	0.0465 (4)	
C16	0.42467 (13)	0.56881 (6)	0.25733 (10)	0.0477 (4)	
C17	0.48294 (15)	0.50353 (6)	0.23145 (10)	0.0536 (4)	
C18	0.44357 (19)	0.46854 (8)	0.13552 (12)	0.0691 (6)	
C19	0.34418 (19)	0.49821 (9)	0.06166 (12)	0.0749 (6)	
C20	0.28575 (18)	0.56245 (10)	0.08314 (12)	0.0736 (6)	
C21	0.32615 (15)	0.59701 (8)	0.17932 (11)	0.0605 (5)	
C22	0.23610 (17)	0.71132 (8)	0.48318 (11)	0.0640 (5)	
C23	0.08391 (19)	0.73546 (10)	0.48559 (15)	0.0822 (7)	
C24	0.0851 (2)	0.79149 (11)	0.32138 (15)	0.0905 (7)	
C25	0.23701 (19)	0.77036 (8)	0.31261 (12)	0.0712 (6)	
H3	0.44030	0.58160	0.42410	0.0530*	
H5	0.60420	0.69530	0.17750	0.0630*	
H6	0.75520	0.68130	0.04450	0.0730*	
H7	0.95760	0.61320	0.07710	0.0780*	
H8	1.01210	0.56180	0.24380	0.0800*	
H11	0.96750	0.54180	0.60040	0.0840*	
H12	0.87440	0.57700	0.75670	0.0800*	
H13	0.67740	0.64990	0.74800	0.0760*	
H14	0.56200	0.67890	0.58290	0.0670*	
H18	0.48450	0.42510	0.12130	0.0830*	
H19	0.31650	0.47480	−0.00280	0.0900*	
H20	0.21860	0.58300	0.03290	0.0880*	
H21	0.28560	0.64080	0.19200	0.0730*	
H22A	0.30050	0.74600	0.51880	0.0770*	
H22B	0.25040	0.66650	0.52080	0.0770*	
H23A	0.02040	0.69950	0.45230	0.0990*	
H23B	0.06300	0.74100	0.56000	0.0990*	
H24A	0.06510	0.83550	0.28260	0.1090*	
H24B	0.02210	0.75530	0.28850	0.1090*	
H25A	0.25350	0.76480	0.23730	0.0850*	
H25B	0.30070	0.80690	0.34390	0.0850*	

### Atomic displacement parameters (Å^2^)

**Table:**

	*U*^11^	*U*^22^	*U*^33^	*U*^12^	*U*^13^	*U*^23^
O1	0.0664 (6)	0.0398 (4)	0.0748 (6)	−0.0032 (4)	0.0010 (5)	−0.0022 (4)
O2	0.0706 (7)	0.0878 (7)	0.0609 (6)	0.0346 (6)	0.0163 (5)	0.0220 (5)
O3	0.0973 (9)	0.0551 (6)	0.0684 (6)	0.0094 (5)	0.0043 (6)	0.0026 (5)
O4	0.1586 (16)	0.1034 (10)	0.1227 (11)	0.0737 (10)	−0.0035 (10)	−0.0381 (8)
O5	0.0755 (8)	0.0936 (8)	0.0909 (8)	0.0321 (6)	0.0160 (6)	−0.0121 (7)
N1	0.0447 (6)	0.0409 (5)	0.0574 (6)	0.0033 (4)	0.0068 (4)	−0.0024 (4)
N2	0.0436 (6)	0.0549 (6)	0.0531 (6)	0.0091 (5)	0.0072 (4)	−0.0030 (5)
N3	0.0818 (9)	0.0435 (6)	0.0781 (9)	0.0063 (6)	0.0150 (7)	−0.0064 (6)
C1	0.0496 (7)	0.0411 (6)	0.0469 (6)	−0.0007 (5)	0.0039 (5)	0.0007 (5)
C2	0.0447 (7)	0.0385 (6)	0.0487 (6)	−0.0007 (5)	0.0057 (5)	0.0010 (5)
C3	0.0455 (7)	0.0391 (6)	0.0496 (6)	0.0004 (5)	0.0093 (5)	−0.0006 (5)
C4	0.0441 (7)	0.0401 (6)	0.0512 (7)	−0.0027 (5)	0.0061 (5)	0.0021 (5)
C5	0.0507 (8)	0.0522 (7)	0.0549 (7)	0.0017 (6)	0.0033 (6)	0.0068 (6)
C6	0.0670 (10)	0.0655 (8)	0.0514 (7)	−0.0037 (7)	0.0104 (7)	0.0098 (6)
C7	0.0629 (9)	0.0732 (9)	0.0613 (8)	0.0010 (7)	0.0224 (7)	0.0042 (7)
C8	0.0559 (9)	0.0780 (9)	0.0680 (9)	0.0149 (7)	0.0182 (7)	0.0120 (7)
C9	0.0512 (8)	0.0549 (7)	0.0557 (7)	0.0056 (6)	0.0097 (6)	0.0095 (6)
C10	0.0553 (8)	0.0580 (7)	0.0533 (7)	0.0044 (6)	0.0079 (6)	0.0104 (6)
C11	0.0618 (10)	0.0788 (10)	0.0678 (9)	0.0127 (7)	0.0022 (7)	0.0209 (8)
C12	0.0745 (11)	0.0693 (9)	0.0543 (8)	−0.0103 (8)	−0.0062 (7)	0.0119 (7)
C13	0.0820 (11)	0.0556 (8)	0.0514 (8)	−0.0096 (7)	0.0054 (7)	−0.0032 (6)
C14	0.0644 (9)	0.0490 (7)	0.0530 (7)	−0.0001 (6)	0.0047 (6)	−0.0043 (6)
C15	0.0483 (7)	0.0411 (6)	0.0498 (7)	−0.0037 (5)	0.0036 (5)	0.0022 (5)
C16	0.0472 (7)	0.0465 (6)	0.0511 (7)	−0.0076 (5)	0.0138 (5)	−0.0034 (5)
C17	0.0606 (8)	0.0454 (6)	0.0569 (7)	−0.0063 (6)	0.0170 (6)	−0.0046 (6)
C18	0.0864 (12)	0.0576 (8)	0.0669 (9)	−0.0111 (8)	0.0263 (8)	−0.0158 (7)
C19	0.0825 (12)	0.0869 (11)	0.0567 (9)	−0.0200 (9)	0.0140 (8)	−0.0232 (8)
C20	0.0652 (10)	0.0960 (12)	0.0588 (9)	−0.0075 (8)	0.0017 (7)	−0.0118 (8)
C21	0.0547 (9)	0.0666 (8)	0.0597 (8)	−0.0003 (6)	0.0029 (6)	−0.0091 (7)
C22	0.0656 (9)	0.0724 (9)	0.0555 (8)	0.0085 (7)	0.0137 (7)	0.0006 (7)
C23	0.0694 (11)	0.1011 (13)	0.0805 (11)	0.0075 (9)	0.0295 (9)	−0.0102 (10)
C24	0.0804 (13)	0.1075 (14)	0.0826 (11)	0.0427 (10)	0.0030 (9)	0.0028 (10)
C25	0.0759 (11)	0.0749 (10)	0.0639 (9)	0.0288 (8)	0.0119 (7)	0.0130 (7)

### Geometric parameters (Å, º)

**Table:**

O1—C1	1.2072 (14)	C16—C17	1.3977 (17)
O2—C9	1.3786 (16)	C16—C21	1.3839 (19)
O2—C10	1.3817 (17)	C17—C18	1.3827 (19)
O3—N3	1.2108 (17)	C18—C19	1.368 (2)
O4—N3	1.214 (2)	C19—C20	1.367 (3)
O5—C23	1.411 (2)	C20—C21	1.383 (2)
O5—C24	1.412 (2)	C22—C23	1.508 (2)
N1—N2	1.4078 (15)	C24—C25	1.500 (3)
N1—C1	1.3546 (16)	C3—H3	0.9800
N1—C3	1.4757 (15)	C5—H5	0.9300
N2—C22	1.4635 (17)	C6—H6	0.9300
N2—C25	1.4655 (18)	C7—H7	0.9300
N3—C17	1.4659 (19)	C8—H8	0.9300
C1—C2	1.5456 (17)	C11—H11	0.9300
C2—C3	1.5989 (17)	C12—H12	0.9300
C2—C4	1.5055 (17)	C13—H13	0.9300
C2—C15	1.5071 (17)	C14—H14	0.9300
C3—C16	1.5084 (17)	C18—H18	0.9300
C4—C5	1.3919 (17)	C19—H19	0.9300
C4—C9	1.3769 (18)	C20—H20	0.9300
C5—C6	1.3761 (19)	C21—H21	0.9300
C6—C7	1.373 (2)	C22—H22A	0.9700
C7—C8	1.370 (2)	C22—H22B	0.9700
C8—C9	1.387 (2)	C23—H23A	0.9700
C10—C11	1.384 (2)	C23—H23B	0.9700
C10—C15	1.3756 (19)	C24—H24A	0.9700
C11—C12	1.371 (2)	C24—H24B	0.9700
C12—C13	1.373 (2)	C25—H25A	0.9700
C13—C14	1.3743 (19)	C25—H25B	0.9700
C14—C15	1.3902 (18)		
			
C9—O2—C10	118.08 (11)	C16—C21—C20	122.45 (14)
C23—O5—C24	109.10 (14)	N2—C22—C23	108.31 (12)
N2—N1—C1	132.68 (10)	O5—C23—C22	111.54 (14)
N2—N1—C3	128.25 (9)	O5—C24—C25	110.85 (14)
C1—N1—C3	97.05 (9)	N2—C25—C24	108.79 (14)
N1—N2—C22	111.11 (10)	N1—C3—H3	112.00
N1—N2—C25	110.09 (10)	C2—C3—H3	112.00
C22—N2—C25	109.78 (10)	C16—C3—H3	112.00
O3—N3—O4	122.62 (15)	C4—C5—H5	119.00
O3—N3—C17	119.36 (12)	C6—C5—H5	119.00
O4—N3—C17	118.02 (14)	C5—C6—H6	120.00
O1—C1—N1	132.94 (12)	C7—C6—H6	120.00
O1—C1—C2	134.74 (12)	C6—C7—H7	120.00
N1—C1—C2	92.26 (9)	C8—C7—H7	120.00
C1—C2—C3	84.86 (9)	C7—C8—H8	120.00
C1—C2—C4	115.94 (9)	C9—C8—H8	120.00
C1—C2—C15	111.77 (9)	C10—C11—H11	120.00
C3—C2—C4	117.12 (9)	C12—C11—H11	120.00
C3—C2—C15	113.79 (9)	C11—C12—H12	120.00
C4—C2—C15	111.09 (10)	C13—C12—H12	120.00
N1—C3—C2	85.81 (8)	C12—C13—H13	120.00
N1—C3—C16	114.57 (9)	C14—C13—H13	120.00
C2—C3—C16	119.27 (10)	C13—C14—H14	119.00
C2—C4—C5	122.55 (11)	C15—C14—H14	119.00
C2—C4—C9	120.18 (10)	C17—C18—H18	120.00
C5—C4—C9	117.26 (11)	C19—C18—H18	120.00
C4—C5—C6	121.69 (12)	C18—C19—H19	120.00
C5—C6—C7	119.47 (13)	C20—C19—H19	120.00
C6—C7—C8	120.49 (14)	C19—C20—H20	120.00
C7—C8—C9	119.32 (14)	C21—C20—H20	120.00
O2—C9—C4	122.59 (11)	C16—C21—H21	119.00
O2—C9—C8	115.65 (12)	C20—C21—H21	119.00
C4—C9—C8	121.76 (12)	N2—C22—H22A	110.00
O2—C10—C11	115.87 (13)	N2—C22—H22B	110.00
O2—C10—C15	122.24 (11)	C23—C22—H22A	110.00
C11—C10—C15	121.89 (13)	C23—C22—H22B	110.00
C10—C11—C12	119.27 (15)	H22A—C22—H22B	108.00
C11—C12—C13	120.38 (14)	O5—C23—H23A	109.00
C12—C13—C14	119.40 (13)	O5—C23—H23B	109.00
C13—C14—C15	121.81 (13)	C22—C23—H23A	109.00
C2—C15—C10	120.43 (11)	C22—C23—H23B	109.00
C2—C15—C14	122.43 (12)	H23A—C23—H23B	108.00
C10—C15—C14	117.13 (12)	O5—C24—H24A	109.00
C3—C16—C17	124.60 (11)	O5—C24—H24B	109.00
C3—C16—C21	119.98 (11)	C25—C24—H24A	109.00
C17—C16—C21	115.42 (12)	C25—C24—H24B	109.00
N3—C17—C16	120.88 (11)	H24A—C24—H24B	108.00
N3—C17—C18	116.45 (12)	N2—C25—H25A	110.00
C16—C17—C18	122.65 (13)	N2—C25—H25B	110.00
C17—C18—C19	119.66 (14)	C24—C25—H25A	110.00
C18—C19—C20	119.61 (14)	C24—C25—H25B	110.00
C19—C20—C21	120.21 (15)	H25A—C25—H25B	108.00
			
C9—O2—C10—C11	−164.66 (13)	C15—C2—C3—C16	−131.34 (11)
C9—O2—C10—C15	15.67 (19)	C15—C2—C4—C5	−160.75 (11)
C10—O2—C9—C4	−16.61 (19)	C1—C2—C4—C9	148.72 (11)
C10—O2—C9—C8	162.73 (13)	C15—C2—C4—C9	19.71 (15)
C23—O5—C24—C25	−61.03 (18)	C15—C2—C3—N1	112.75 (10)
C24—O5—C23—C22	60.87 (18)	C2—C3—C16—C17	70.79 (16)
N2—N1—C1—O1	−11.8 (2)	C2—C3—C16—C21	−108.37 (14)
C3—N1—N2—C25	−149.25 (11)	N1—C3—C16—C21	−8.93 (17)
C1—N1—N2—C22	−71.02 (15)	N1—C3—C16—C17	170.23 (11)
C3—N1—N2—C22	88.92 (13)	C5—C4—C9—C8	−1.01 (19)
N2—N1—C3—C2	−166.66 (11)	C2—C4—C5—C6	−179.05 (12)
C1—N1—C3—C2	−1.38 (9)	C2—C4—C9—C8	178.56 (12)
N2—N1—C3—C16	72.97 (14)	C5—C4—C9—O2	178.30 (12)
C3—N1—C1—O1	−176.04 (14)	C2—C4—C9—O2	−2.14 (19)
N2—N1—C1—C2	165.68 (11)	C9—C4—C5—C6	0.51 (19)
C1—N1—N2—C25	50.80 (16)	C4—C5—C6—C7	0.5 (2)
C3—N1—C1—C2	1.42 (9)	C5—C6—C7—C8	−0.9 (2)
C1—N1—C3—C16	−121.74 (11)	C6—C7—C8—C9	0.5 (2)
C22—N2—C25—C24	−58.02 (15)	C7—C8—C9—C4	0.6 (2)
N1—N2—C22—C23	179.19 (11)	C7—C8—C9—O2	−178.80 (13)
C25—N2—C22—C23	57.18 (16)	C11—C10—C15—C14	3.1 (2)
N1—N2—C25—C24	179.37 (12)	O2—C10—C11—C12	178.81 (14)
O3—N3—C17—C18	−159.81 (14)	O2—C10—C15—C14	−177.26 (12)
O4—N3—C17—C16	−161.84 (14)	C11—C10—C15—C2	−175.75 (13)
O3—N3—C17—C16	18.9 (2)	O2—C10—C15—C2	3.91 (19)
O4—N3—C17—C18	19.4 (2)	C15—C10—C11—C12	−1.5 (2)
N1—C1—C2—C4	116.42 (11)	C10—C11—C12—C13	−1.7 (2)
N1—C1—C2—C15	−114.90 (10)	C11—C12—C13—C14	3.1 (2)
O1—C1—C2—C3	176.08 (14)	C12—C13—C14—C15	−1.5 (2)
N1—C1—C2—C3	−1.31 (8)	C13—C14—C15—C2	177.22 (12)
O1—C1—C2—C4	−66.19 (18)	C13—C14—C15—C10	−1.6 (2)
O1—C1—C2—C15	62.49 (18)	C3—C16—C17—C18	179.68 (13)
C1—C2—C3—N1	1.20 (8)	C17—C16—C21—C20	1.2 (2)
C4—C2—C3—N1	−115.37 (10)	C21—C16—C17—N3	−179.79 (12)
C1—C2—C3—C16	117.11 (10)	C3—C16—C17—N3	1.0 (2)
C4—C2—C3—C16	0.54 (15)	C21—C16—C17—C18	−1.1 (2)
C1—C2—C4—C5	−31.73 (16)	C3—C16—C21—C20	−179.54 (13)
C4—C2—C15—C10	−20.60 (16)	N3—C17—C18—C19	178.99 (14)
C4—C2—C15—C14	160.64 (11)	C16—C17—C18—C19	0.3 (2)
C3—C2—C4—C9	−113.39 (13)	C17—C18—C19—C20	0.6 (3)
C3—C2—C4—C5	66.16 (15)	C18—C19—C20—C21	−0.5 (3)
C1—C2—C15—C10	−151.80 (12)	C19—C20—C21—C16	−0.5 (2)
C1—C2—C15—C14	29.43 (16)	N2—C22—C23—O5	−59.13 (17)
C3—C2—C15—C10	114.15 (13)	O5—C24—C25—N2	59.98 (18)
C3—C2—C15—C14	−64.62 (15)		

### Hydrogen-bond geometry (Å, º)

**Table:**

*D*—H···*A*	*D*—H	H···*A*	*D*···*A*	*D*—H···*A*
C3—H3···O3^i^	0.98	2.55	3.5310 (16)	174
C6—H6···O1^ii^	0.93	2.56	3.3828 (17)	148
C11—H11···O2^iii^	0.93	2.50	3.389 (2)	159

Symmetry codes: (i) −*x*+1, −*y*+1, −*z*+1; (ii) *x*, −*y*+3/2, *z*−1/2; (iii) −*x*+2, −*y*+1, −*z*+1.
